# Fenofibrate Inhibits Subretinal Fibrosis Through Suppressing TGF‐β—Smad2/3 signaling and Wnt signaling in Neovascular Age‐Related Macular Degeneration

**DOI:** 10.3389/fphar.2020.580884

**Published:** 2020-11-17

**Authors:** Qian Chen, Nan Jiang, Yuhan Zhang, Sihao Ye, Xu Liang, Xin Wang, Xiang Lin, Rongrong Zong, Haoyu Chen, Zuguo Liu

**Affiliations:** ^1^ Department of Ophthalmology, Xiang’an Hospital of Xiamen University, Fujian Provincial Key Laboratory of Ophthalmology and Visual Science, Eye Institute of Xiamen University, School of Medicine, Xiamen University, Xiamen, China; ^2^ Xiamen University affiliated Xiamen Eye Center, Xiamen, China; ^3^ Joint Shantou International Eye Center of Shantou University and the Chinese University of Hong Kong, Shantou, China

**Keywords:** Fenofibrate, subretinal fibrosis, neovascular age-related macular degeneration, Wnt signaling, connective tissue growth factor, very low‐density lipoprotein receptor

## Abstract

Subretinal fibrosis is a common pathological change that causes vision loss in neovascular age-related macular degeneration (nAMD). Treatment modalities for subretinal fibrosis are limited. In the present study, the effects of fenofibrate, a specific peroxisome proliferator–activated receptor alpha agonist, on subretinal fibrosis of nAMD were tested, and its molecular mechanisms of action were delineated. Collagen deposition and protein expression of fibrotic markers, such as vimentin, collagen-1, alpha-smooth muscle actin, and fibronectin, were increased in very low–density lipoprotein receptor (VLDLR) knockout mouse, indicating *Vldlr* ^*−/−*^ mice can be used as a model for subretinal fibrosis. Fenofibrate suppressed subretinal fibrosis of *Vldlr* ^*−/−*^ mice by reducing collagen deposition and protein expression of fibrotic markers. Two fibrotic pathways, TGF-β—Smad2/3 signaling and Wnt signaling, were significantly up-regulated, while inhibited by fenofibrate in *Vldlr* ^*−/−*^ retinas. Moreover, fenofibrate significantly reduced the downstream connective tissue growth factor (CTGF) expression of these two pathways. Müller cells were a major source of CTGF in *Vldlr* ^*−/−*^ retinas. Fenofibrate was capable of suppressing Müller cell activation and thus reducing the release of CTGF in *Vldlr* ^*−/−*^ retinas. In cultured Müller cells, fenofibrate reversed TGF-β2–induced up-regulation of Wnt signaling and CTGF expression. These findings suggested that fenofibrate inhibits subretinal fibrosis by suppressing TGF-β—Smad2/3 signaling and Wnt signaling and reducing CTGF expression, and thus, fenofibrate could be a potential treatment for nAMD with subretinal fibrosis.

## Introduction

Age-related macular degeneration (AMD) is a leading cause of blindness in the elderly of Western societies (Lim et al., 2012). Neovascular AMD (nAMD), also called “Wet AMD,” is a type of AMD, which is characterized by choroid new neovascularization (CNV) (Ishikawa et al., 2016). Recently, anti-VEGF drugs have been standardized for the treatment of nAMD. However, more than 10% patients are unresponsive to anti-VEGF treatment and may progress to subretinal fibrous scar formation and massive subretinal hemorrhage (Kovach et al., 2012). The process of subretinal fibrosis involves excessive response to tissue damage, proliferation, and infiltration of various cell types, including retinal pigment epithelial (RPE) cells, glial cells, macrophages, and fibroblasts, and formation of extracellular matrix (ECM) (Ishikawa et al., 2016). The formation of subretinal fibrosis disrupts the physical connections of retinal cells and results in abnormal functioning of retina, and even loss of function (Ishikawa et al., 2016). Currently, the molecular mechanisms of subretinal fibrosis are still unclear, and the clinical treatment for subretinal fibrosis is very limited, especially for the late-stage nAMD.

Fenofibrate, a specific peroxisome proliferator–activated receptor alpha (PPARα) agonist, is widely used in clinic to control the blood lipid levels (Brown, 2007). Two clinical trials have identified the therapeutic effects of fenofibrate on late-stage diabetic retinopathy (DR) in patients with type 2 diabetes (Keech et al., 2007). The mechanisms of fenofibrate’s protective effects in DR are reported to protect retinal cells from oxidative stress and apoptosis, improve retinal neuronal function, and reduce retinal inflammation (Ding et al., 2014; Moran et al., 2014; Pearsall et al., 2017; Qiu et al., 2017).

Recently, the anti-fibrotic effects of fenofibrate have been reported in renal and cardiovascular systems. For instance, a study showed that fenofibrate attenuated the expression of fibrotic factors in the kidney of both type 1 and type 2 diabetic models (Cheng et al., 2016). Zhang et al. reported that fenofibrate prevented fibrosis and inflammation in the heart of streptozotocin-induced diabetic rats (Zhang et al., 2016) . In addition, fenofibrate is reported to reduce fibroblast myofibroblast transition and subepithelial fibrosis in asthmatic patients (Paw et al., 2018). However, it is still unknown whether fenofibrate has anti-fibrotic effects in the retina.

Studies have reported that the TGF-β—Smad2/3 signaling pathway and the Wnt signaling pathway are the most common pathways that participate in the formation and development of tissue fibrosis (Meng et al., 2016; Burgy and Konigshoff, 2018). The roles of TGF-β—Smad2/3 signaling in subretinal firbosis of nAMD have been well studied (Tosi et al., 2018). However, the involvement of the Wnt signaling pathway in retinal fibrosis is still unclear, especially in the late-stage nAMD. Studies have shown that fenofibrate was capable of inhibiting Wnt signaling in the kidney (Cheng et al., 2016). It is possible that fenofibrate may also inhibit Wnt signaling in the retina. Interestingly, connective tissue growth factor (CTGF), a factor that leads to fibrosis in multiple tissues, has been reported to be a downstream target of these two pathways (Luo et al., 2004; Lipson et al., 2012; Liu et al., 2013). CTGF is highly present in the human proliferative vitreoretinopathy (PVR) membrane and associated with intraocular fibrosis (He et al., 2008). The investigation of profibrotic signaling pathways and CTGF may shed some light on the mechanism of formation of subretinal fibrosis in nAMD. Additionally, the exploration of pharmacological treatment for inhibiting CTGF may be beneficial to patients with retinal fibrosis.

In this study, we identified that the presence of subretinal fibrosis in the retina of an nAMD animal model—very low–density lipoprotein knockout (*Vldlr* ^*−/−*^) mice. Then, the effects of fenofibrate on subretinal fibrosis were tested in the retina of *Vldlr* ^*−/−*^ mice. The expressions of fibrotic TGF-β—Smad2/3 signaling and Wnt signaling were measured, and their downstream target CTGF was evaluated in the retina of *Vldlr* ^*−/−*^ mice. Moreover, the inhibitory effects of fenofibrate on the two signaling pathways and CTGF were tested. Further, we have explored the major source of CTGF and found that fenofibrate suppressed this source of CTGF in *Vldlr* ^*−/−*^ retinas. Our study demonstrated that *Vldlr* ^*−/−*^ mice could be an animal model of subretinal fibrosis and explored a possible new application of fenofibrate in treating nAMD with subretinal fibrosis.

## Materials and Methods

### Animals

B6; 129S7-Vldlr^tm1Her^/J (*Vldlr* ^*−/−*^) mice were obtained from the Jackson Laboratory (Bar Harbor, ME). Age-matched wild-type (WT) mice at a similar genetic background were obtained from crossing C57BL/6J and *Vldlr* ^*−/−*^ mice. All the mice were raised in the laboratory animal center of Xiamen University (Xiamen, Fujian, China). The animal experiments were performed according to the ARVO Statement for the Use of Animals in Ophthalmic and Vision Research, and all studies were conducted in accordance with a protocol approved by the Experimental Animal Ethics Committee of Xiamen University.

### *In Vivo* Experimental Procedure

The special animal diet containing 0.1% fenofibrate (Sigma-Aldrich, MO, United States) and control diet were purchased from Medicine Biomedicine Co. Ltd. (Jiangsu, Zhejiang, China). Three-month-old *Vldlr* ^*−/−*^ mice and littermate control WT mice were fed with the diet containing fenofibrate. After 45 days of feeding, the mice were sacrificed, and their eyecups were harvested. Similarly, seven-month-old *Vldlr* ^*−/−*^ mice and control mice were fed with the diet containing fenofibrate for 60 days, and their eyes were collected for further experiments.

### Cell Culture Procedures

The rat Müller (rMC-1) cell line is a generous gift from Dr. Vijay Sarthy of Northwestern University, United States, and maintained in Dulbecco’s modified Eagle medium basal medium containing 10% fetal bovine serum (Gibco, Thermo Fisher Scientific, Waltham, MA, United States) and 10% penicillin/streptomycin solution (Gibco) at 37°C in humidified atmosphere (95% air, 5% CO_2_).

### Quantitative Real-Time RT-PCR

Total RNA was extracted from the eyecups with reagent (TRIzol; Invitrogen). 500 ng of total RNA was reverse-transcribed into complementary DNA (cDNA) using a ReverTra Ace qPCR RT Master Mix (Toyobo, Osaka, Japan). Quantitative real-time PCR was performed using the Hieff™ qPCR SYBR Green Master Mix (Yeasen, Shanghai, China) in the StepOne Real-Time PCR System (Thermo Fisher Scientific) according to the manufacturer’s instructions. qPCR primers were synthesized by Sangon Biotech (Shanghai, China). The set of primers GFAP (forward primer, 5′-ATC GAG ATC GCC ACC TAC AG-3′; and reverse primer, 5′-TAC CAC GAT GTT CCT CTT GA-3′) and CTGF (forward primer, 5′-GTG CCA GAA CGC ACA CTG-3′, and reverse primer, 5′-CCC CGG TTA CAC TCC AAA-3′) were used, and the mRNA levels were normalized to those of glyceraldehyde-3-phosphate dehydrogenase (GAPDH) (forward primer, 5′-ACC ACG AGA AAT ATG ACA ACT CCC-3′; and reverse primer, 5′-CCA AAG TTG TCA TGG ATG ACC-3′) levels.

### Western Blot Analysis

The eyecups were lysed in radioimmunoprecipitation assay buffer. An equal amount of total proteins was subjected to electrophoresis on 10% SDS-PAGE and then electrophoretically transferred to a polyvinylidene fluoride film. The member was blocked with 10% non-fat milk for 2 h at room temperature (RT) and incubated with primary antibody overnight at 4°C. After several washes, the membrane was then incubated with secondary antibody for 1 h at RT. The signal was developed with an enhanced chemiluminescence reagent kit (NCM Biotech, Newport, RI, United States). The bands were quantified with an ImageJ density analyzer and normalized to GAPDH levels. Antibodies for vimentin, collagen-1, alpha-smooth muscle actin (α-SMA), and CTGF were purchased from Abcam (Cambridge, United Kingdom). Antibodies for fibronectin, Smad2/3, and TGF-β receptor 2 (TGF-βR2) were obtained from Santa Cruz Biotechnology (Dallas, TX, United States). Antibodies for glycogen synthase kinase 3 beta (GSK3β), phosphorylated-GSK3β (p-GSK3β), phosphorylated-Smad2/3 (p-Smad2/3), anti-non–phosphorylated β-catenin (non–p-β-catenin), and GAPDH were purchased from Cell Signaling Technology (Danvers, MA, United States).

### Immunofluorescent Staining

The eyecups were fixed with 4% paraformaldehyde and embedded in optimal cutting temperature compound. Frozen sections of 10 μm thickness were fixed in cold acetone (−20°C) for 10 min. Sections were incubated with 0.2% Triton X-100 for 20 min and blocked with 2% BSA in PBS for 60 min. Then, sections were incubated with different primary antibodies for 16 h at 4°C. After washing three times, sections were incubated with Alexa Fluor 594-conjugated IgG (Abcam) or Alexa Fluor 488-conjugated IgG (Abcam) for 60 min at 37°C. The nucleus was counterstained with 4′, 6-diabmidino-2-phenylindole (Abcam). Images were acquired using a Leica microscope system (DM2500, Wetzlar, Germany).

### Statistical Analysis

All the experiments were performed at least three times. Statistical data were expressed as mean ± SEM. Prism 6 (GraphPad, San Diego, CA, United States) was used to calculate a multivariable ordinary one-way ANOVA or Student’s *t*-test for statistical analysis. *p* < 0.05 was considered statistically significant.

## Results

### Identification of Very Low–Density Lipoprotein Receptor^*−/−*^ Mouse as a Model of Subretinal Fibrosis

**FIGURE 1:**
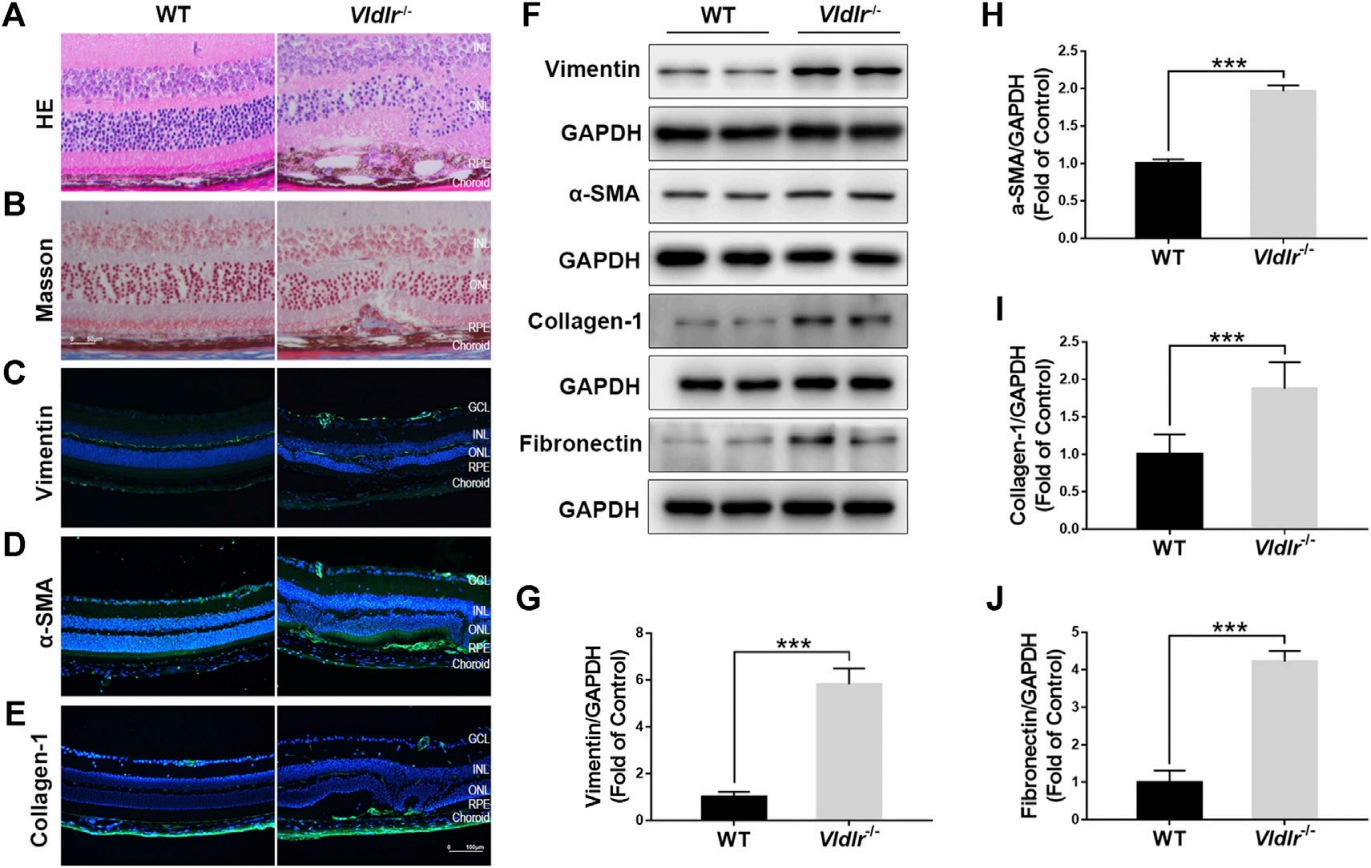
Presence of subretinal fibrosis in very low–density lipoprotein receptor (*Vldlr*)^*−/−*^ retinas. **(A)** Representative H&E staining of eyecups from wild-type (WT) and *Vldlr* ^*−/−*^ mice. **(B)** Masson staining of collagen (blue) in the eyecups of WT mice and *Vldlr* ^*−/−*^ mice. Representative images of immunostaining of **(C)** vimentin, **(D)** alpha-smooth muscle actin (α-SMA), and **(E)** collagen-1 in the eyecups of WT mice and *Vldlr* ^*−/−*^ mice. **(F)** Protein levels of vimentin, α-SMA, collagen-1, and fibronectin in the eyecups of WT mice and *Vldlr* ^*−/−*^ mice were measured by Western blot analysis. Levels of **(G)** vimentin, **(H)** α-SMA, **(I)** collagen-1, and **(J)** fibronectin were quantified by densitometry and normalized to GAPDH levels (mean ± SEM; n = 8. ***p < 0.001).

To test the effects of fenofibrate on subretinal fibrosis, an appropriate animal model of subretinal fibrosis is needed. Currently, the animal models of subretinal fibrosis are limited. *Vldlr* ^*−/−*^ mouse has been reported as a model of nAMD (Hu et al., 2008; Joyal et al., 2016); thus, we checked whether *Vldlr* ^*−/−*^ mice could be used as an animal model of subretinal fibrosis. HE staining of retinal sections of 3-month-old *Vldlr* ^*−/−*^ mice showed that retinal vessels grew into the subretinal area, with infiltrated dark pigment cells (Figure 1A). Masson staining showed the blue collagen deposited into the subretinal area (Figure 1B) in *Vldlr* ^*−/−*^ retinas. Immunostaining signals of fibrotic markers vimentin (Figure 1C), collagen-1 (Figure 1D), and α-SMA (Figure 1E) were significantly up-regulated in the cryosections of *Vldlr* ^*−/−*^ retinas compared to those of WT retinas. In addition, protein levels of vimentin (Figures 1F,G), α-SMA (Figures 1F,H), collagen-1 (Figures 1F,I), and fibronectin (Figures 1,J) were significantly up-regulated in *Vldlr* ^*−/−*^ retinas compared with WT retinas. In addition, Masson staining of collagen fiber was performed in 2-, 3-, 5-, and 8-month-old *Vldlr* ^*−/−*^ retinas, respectively. The results indicated that collagen fibers started to appear in the subretinal area of *Vldlr* ^*−/−*^ mice at the age of 2 months, reach a substantial level at 3 months , and persistent in the mice over the age of 3 months (Supplementary Figure S1). Taken together, these results indicate the presence of subretinal fibrosis in the retina of *Vldlr* ^*−/−*^ mice, which could be used as a mouse model of subretinal fibrosis.

**FIGURE 2:**
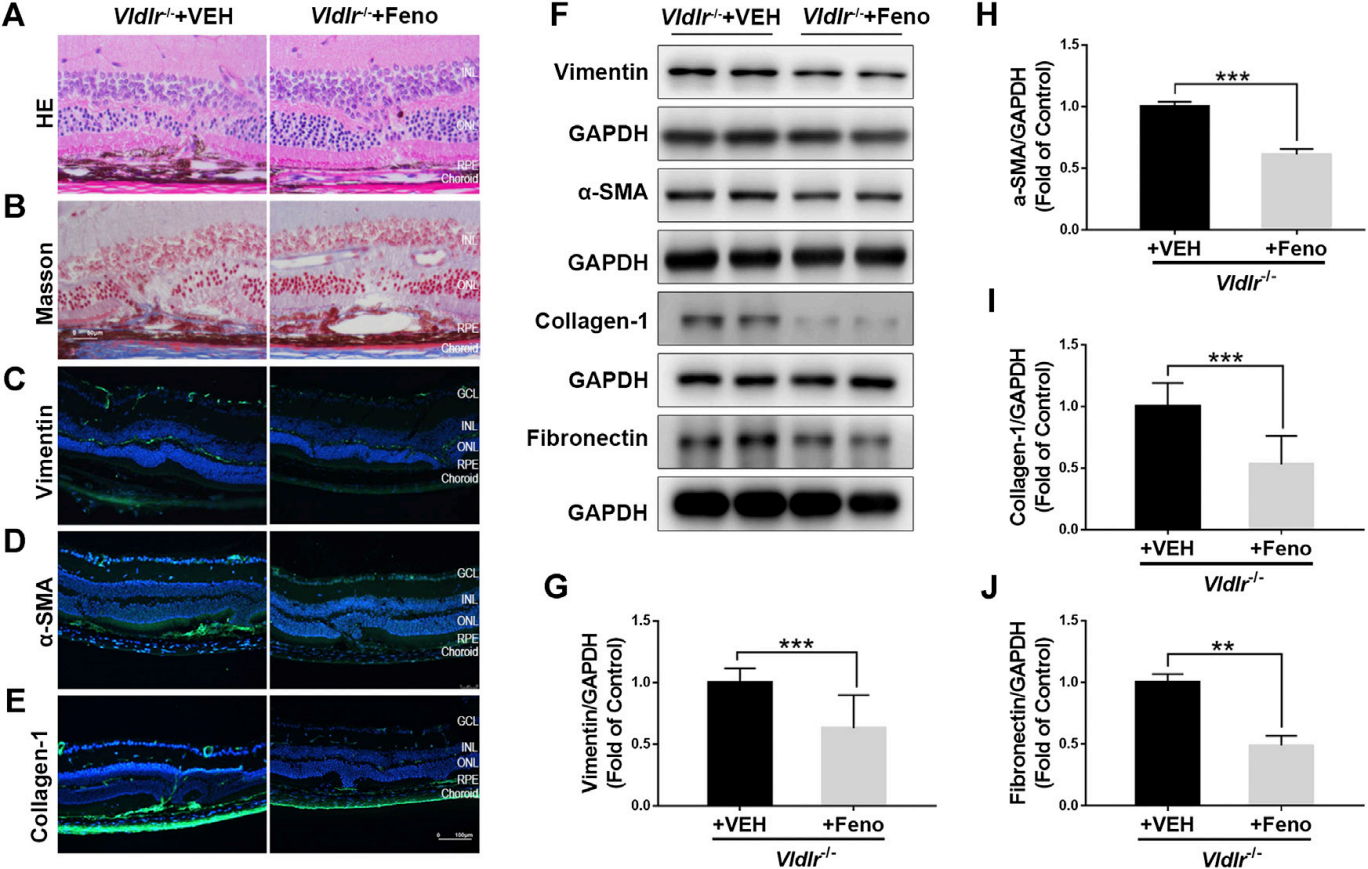
Fenofibrate ameliorates subretinal fibrosis in very low–density lipoprotein receptor (*Vldlr)* ^*−/−*^ mice. **(A)** Representative retinal images of H&E staining from *Vldlr* ^*−/−*^ mice fed with control chow [*Vldlr* ^*−/−*^ + vehicle (VEH)] or fenofibrate chow (*Vldlr* ^*−/−*^ + feno). **(B)** Representative images of Masson staining of collagen deposition in *Vldlr* ^*−/−*^ mice fed with control chow (*Vldlr* ^*−/−*^ + VEH) or fenofibrate chow (*Vldlr* ^*−/−*^ + feno). Representative images of immunohistochemistry show the expression of **(C)** vimentin, **(D)** alpha-smooth muscle actin (α-SMA), and **(E)** collagen-1 in the eyecups of *Vldlr* ^*−/−*^ + VEH or *Vldlr* ^*−/−*^ + feno mice. Levels of **(F,G)** vimentin, **(F,H)** α-SMA, **(F,I)** collagen-1, and **(F,J)** fibronectin were determined by Western blot analysis and quantified by densitometry in the eyecups of *Vldlr* ^*−/−*^ + VEH or *Vldlr* ^*−/−*^ + feno mice (mean ± SEM; n = 8. **p < 0.01 and ***p < 0.001).

### Fenofibrate Reverses the Up-Regulation of Fibrotic Markers in Very Low–Density Lipoprotein Receptor^*−/−*^ Retinas

Next, *Vldlr* ^*−/−*^ mice were fed with fenofibrate-containing chow or normal (control) chow. To test whether oral administration of fenofibrate was effective in the retina, we measured the protein expression of fatty acyl-CoA oxidase 1 (ACOX1), a downstream target gene of PPARα (Fan et al., 1998). Levels of ACOX1 were significantly up-regulated in the retina of *Vldlr* ^*−/−*^ mice fed with fenofibrate chow compared to those fed with vehicle (VEH), suggesting that the effects of fenofibrate were achieved in the retina (Supplementary Figure S2). HE staining showed fewer infiltrated dark pigment cells in the subretinal area of *Vldlr* ^*−/−*^ mice fed with fenofibrate (Figure 2A). Similarly, less collagen deposition was observed in the retina of *Vldlr* ^*−/−*^ mice fed with fenofibrate chow (Figure 2B). Immunostaining of vimentin (Figure 2C), collagen-1 (Figure 2D), and α-SMA (Figure 2E) showed less immunofluorescent signals in the retina of *Vldlr* ^*−/−*^ mice treated with fenofibrate than those treated with VEH. Further, protein levels of vimentin (Figures 2F,G), α-SMA (Figures 2F,H), collagen-1 (Figures 2F,I), and fibronectin (Figures 2F,J) were significantly decreased in the retina of *Vldlr* ^*−/−*^ mice treated with fenofibrate. Taken together, these results suggest that fenofibrate suppresses subretinal fibrosis in the context of *Vldlr* ^*−/−*^ mice.

### Suppression of TGF-β—Smad2/3 Signaling by Fenofibrate in the Retina of *Vldlr* ^*−/−*^ Mice

**FIGURE 3:**
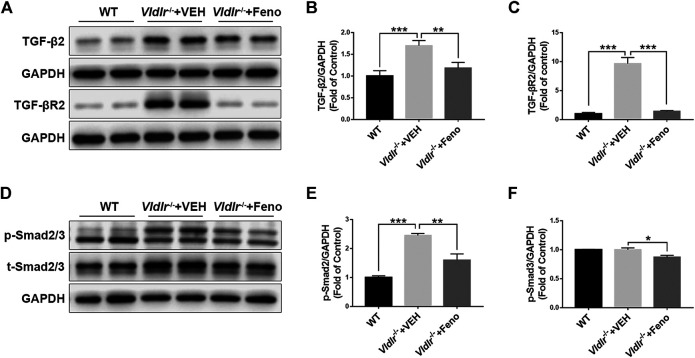
Fenofibrate suppresses TGF-β/Smad2/3 signaling in very low–density lipoprotein receptor (*Vldlr*)^*−/−*^ retinas. Protein levels of **(A,B)** TGF-β2, **(A C)** TGF-βR2, **(D,E)** p-Smad2/3, and **(D,F)** t-Smad2/3 were measured by Western blot analysis and quantified by densitometry in eyecups of wild-type mice and *Vldlr* ^*−/−*^ mice fed with control chow (*Vldlr* ^*−/−*^ + vehicle) or fenofibrate chow (*Vldlr* ^*−/−*^ + feno) (mean ± SEM; n = 8. *p < 0.05, **p < 0.01 and ***p < 0.001).

TGF-β—Smad2/3 signaling is a classic fibrotic signaling that promotes tissue fibrosis in multiple diseases such as liver fibrosis, lung fibrosis, and retinal fibrosis (Hu et al., 2018). To test the mechanism of fenofibrate in inhibiting subretinal fibrosis, key components of the TGF-β—Smad2/3 signaling pathway, TGF-β2, TGF-βR2, p-Smad2/3, and total Smad2/3 (t-Smad2/3) were measured by Western blot analysis (Figures 3A,D). The results showed that protein expressions of TGF-β2 (Figure 3B), TGF-βR2 (Figure 3C), p-Smad2/3 (Figure 3E), and t-Smad2/3 (Figure 3F) were significantly up-regulated in *Vldlr* ^*−/−*^ retinas, further supporting that retinal fibrosis was present in *Vldlr* ^*−/−*^ mice. Fenofibrate treatment reversed the up-regulation of TGF-β2 (Figure 3B), TGF-βR2 (Figure 3C), p-Smad2/3 (Figure 3E), and t-Smad2/3 (Figure 3F) in *Vldlr* ^*−/−*^ retinas, suggesting inhibition of TGF-β—Smad2/3 signaling by fenofibrate.

### Fenofibrate Inhibits Wnt Signaling in the Retina of Very Low–Density Lipoprotein Receptor^*−/−*^ Mice

**FIGURE 4:**
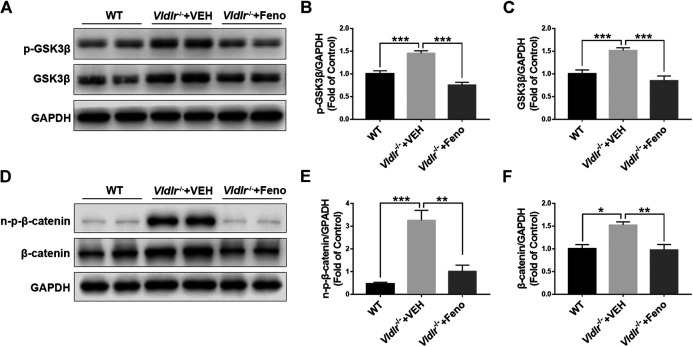
Fenofibrate inhibits Wnt signaling in very low–density lipoprotein receptor (*Vldlr*)^*−/−*^ retinas. Levels of **(A,B)** p-glycogen synthase kinase 3 beta (GSK3β), **(A,C)** GSK3β, **(D,E)** non–p-β-catenin, and **(D,F)** β-catenin were determined by Western blot analysis and quantified by densitometry in eyecups of wild-type mice and *Vldlr* ^*−/−*^ mice fed with control chow (*Vldlr* ^*−/−*^ + vehicle) or fenofibrate chow (*Vldlr* ^*−/−*^ + feno) (mean ± SEM; n = 8. **p < 0.01 and ***p < 0.001).

Wnt signaling is an important signaling pathway that contributes to tissue fibrosis (Burgy and Konigshoff, 2018). Previous studies have shown that Wnt signaling was aberrantly activated in *Vldlr* ^*−/−*^ mice (Chen et al., 2007). Our results also showed that components of Wnt signaling, p-GSK3β (Figures 4A,B), GSK3β (Figures 4A,C), non–p-β-catenin (Figures 4D,E), and β-catenin (Figures 4D,F), were significantly up-regulated in *Vldlr* ^*−/−*^ retinas. In addition, the up-regulated Wnt signaling components, p-GSK3β (Figures 4A, B), GSK3β (Figures 4A,C), non–p-β-catenin (Figures 4D,E), and β-catenin (Figures 4D,F), were significantly reversed in *Vldlr* ^*−/−*^ retinas treated with fenofibrate compared to those treated with VEH, suggesting that fenofibrate suppresses Wnt signaling in the context of *Vldlr* ^*−/−*^ retinas.

### Fenofibrate Suppresses Connective Tissue Growth Factor Expression in the Retina of Very Low–Density Lipoprotein Receptor^*−/−*^ Mice

**FIGURE 5:**
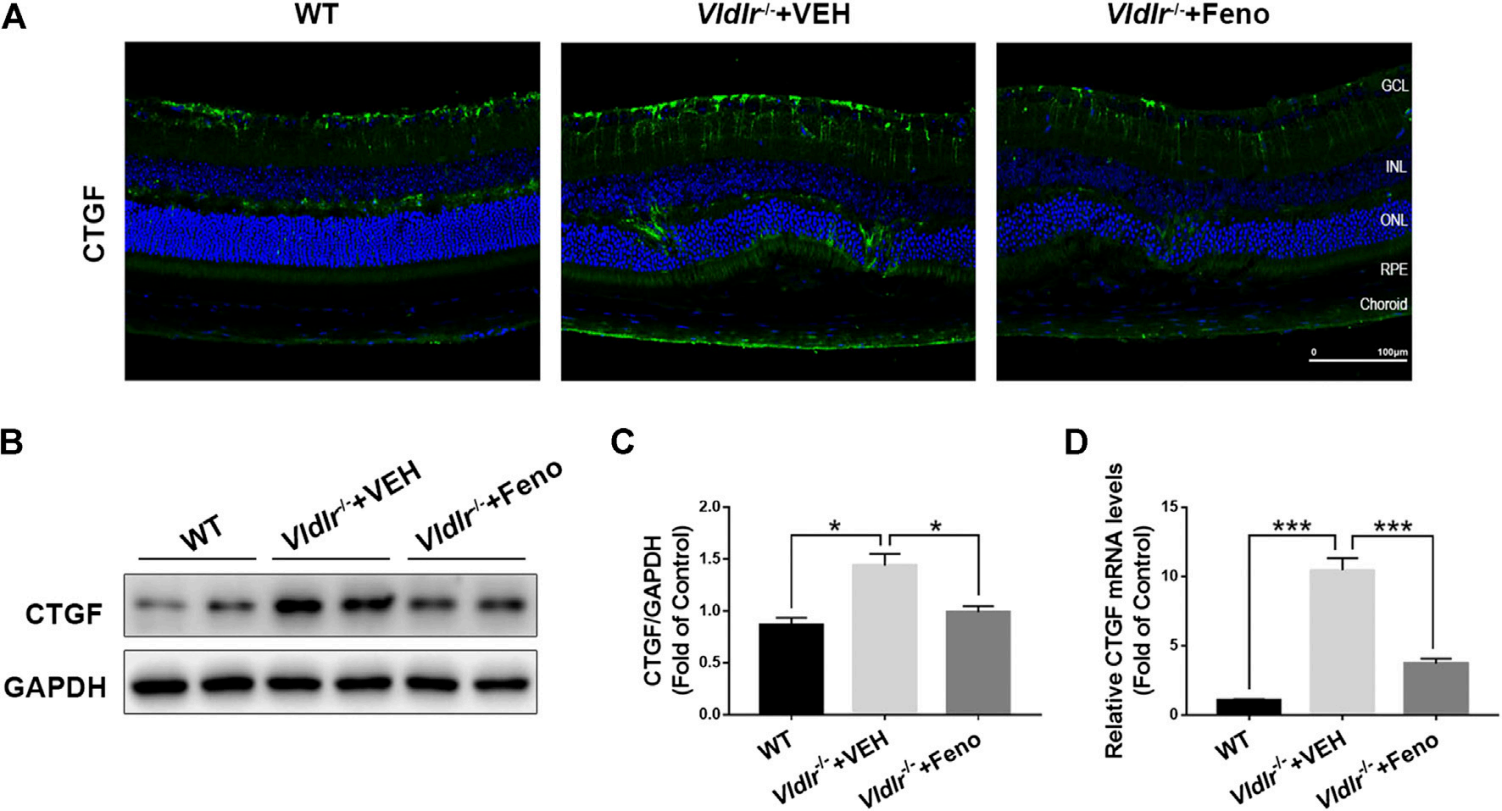
Fenofibrate reduces connective tissue growth factor (CTGF) expression in very low–density lipoprotein receptor (*Vldlr*)^*−/−*^ retinas. **(A)** Representative images of immunofluorescence of CTGF in the eye of wild-type mice and *Vldlr* ^*−/−*^ mice fed with control chow (*Vldlr* ^*−/−*^ + vehicle) or fenofibrate chow (*Vldlr* ^*−/−*^ + feno). **(B,C)** Protein levels of CTGF in the three groups were determined by Western blot analysis and quantified by densitometry. **(D)** Real-time RT-PCR was performed to quantify relative mRNA levels of CTGF in the three groups (mean ± SEM; n = 8. **p < 0.01 and ***p < 0.001).

CTGF, which is elevated in the retina with fibrotic changes, has been considered to be a major fibrotic cytokine that promotes retinal fibrosis (He et al., 2008). Multiple studies have showed that CTGF is a downstream factor of TGF-β—Smad2/3 signaling and Wnt signaling (Luo et al., 2004; Kita et al., 2007; Liu et al., 2013). Therefore, we tested the protein expression of CTGF in control retinas and *Vldlr* ^*−/−*^ retinas which were shown to have subretinal fibrosis in the above figures. The immunofluorescent signal of CTGF (green) was much stronger in the retinal sections of *Vldlr* ^*−/−*^ mice than those in WT mice (Figure 5D), indicating the up-regulation of CTGF expression in *Vldlr* ^*−/−*^ retinas. Whereas less CTGF immunofluorescent signal was observed in the retina of *Vldlr* ^*−/−*^ mice treated with fenofibrate (Figure 5D). Additionally, both mRNA and protein levels of CTGF were elevated; while fenofibrate suppressed the elevated CTGF at mRNA and protein levels in *Vldlr* ^*−/−*^ retinas (Figures 5A–C). Taken together, these results indicate that CTGF is up-regulated in *Vldlr* ^*−/−*^ retinas, and fenofibrate reduces the CTGF expression in *Vldlr* ^*−/−*^ retinas.

### Inhibition of Müller-Secreted Connective Tissue Growth Factor and Müller Gliosis by Fenofibrate in Very Low–Density Lipoprotein Receptor^*−/−*^ Retinas

**FIGURE 6:**
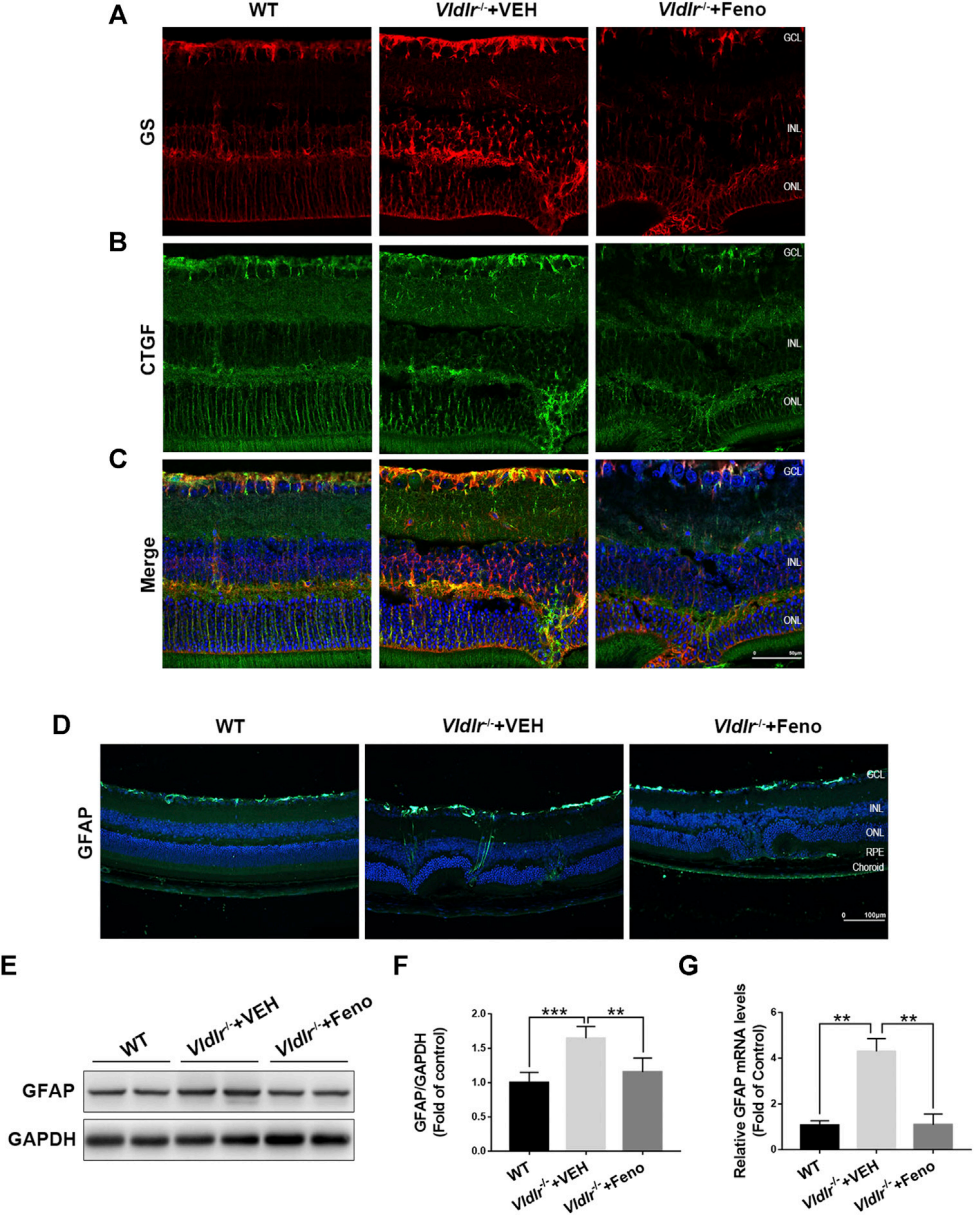
Fenofibrate inhibits Müller-secreted connective tissue growth factor (CTGF) and Müller gliosis in very low–density lipoprotein receptor (*Vldlr*)^*−/−*^ retinas. **(A)** Representative images of immunofluorescence of glutamine synthetase (GS) (red) in the eye of wild-type mice and *Vldlr* ^*−/−*^ mice fed with control chow (*Vldlr* ^*−/−*^ + vehicle) or fenofibrate chow (*Vldlr* ^*−/−*^ + feno). **(B)** Representative images of immunofluorescence of CTGF (green) in the retinas of the indicated three groups. **(C)** The immunofluorescent signals of GS and CTGF were merged. **(D)** Representative images of immunofluorescence of GFAP, a Müller glial activation marker, in the retinas of the indicated three groups. **(E,F)** The protein expression of GFAP in the eyecups of indicated three groups were determined by Western blot analysis and quantified by densitometry. **(G)** Real-time RT-PCR was performed to quantify relative retinal mRNA levels of GFAP in the indicated three groups (mean ± SEM; n = 8. *p < 0.05 and ***p < 0.001).

Next, we determined to identify the source of CTGF and the mechanisms by which fenofibrate suppressed CTGF expression in *Vldlr* ^*−/−*^ retinas. Müller cells play pathogenic roles in retinal fibrosis by secreting multiple factors (Friedlander, 2007). Thus, we investigated whether Müller cells were one of the sources of CTGF. In the retina, glutamine synthetase (GS) is exclusively expressed in Müller cells (Germer et al., 1997). Immunofluorescence of GS (red) was much stronger in *Vldlr* ^*−/−*^ retinas than WT retinas, indicating the activation and/or proliferation of Müller cells in *Vldlr* ^*−/−*^ retinas (Figure 6A). Fenofibrate suppressed Müller cell activation and/or proliferation, as indicated by reduced GS immunofluorescence (Figure 6A). Compared to WT retinas, immunofluorescence of CTGF was up-regulated in *Vldlr* ^*−/−*^ retinas, while fenofibrate reduced the immunofluorescence of CTGF (Figure 6B). The merged immunofluorescent signals of GS (red) and CTGF (green) showed Müller cells were colocalized with CTGF (Figure 6C), suggesting Müller cells were a major source of CTGF in *Vldlr* ^*−/−*^ retinas. In addition, immunostaining of the glial cell activation marker, GFAP, showed that immunofluorescence of GFAP was much stronger in *Vldlr* ^*−/−*^ retinas than WT retinas and became less in *Vldlr* ^*−/−*^ retinas treated with fenofibrate than those treated VEH, suggesting Müller cells were activated, while fenofibrate inhibited the activation of Müller cells in *Vldlr* ^*−/−*^ retinas (Figure 6D). Moreover, both mRNA and protein levels of GFAP were up-regulated in *Vldlr* ^*−/−*^ retinas, whereas fenofibrate suppressed the up-regulated GFAP at mRNA and protein levels (Figure 6E‐G). Taken together, these results suggest that fenofibrate suppresses Müller cell activation and thus reduces CTGF expression in *Vldlr* ^*−/−*^ retinas.

### Fenofibrate Inhibits TGF-β2–Induced Up-Regulation of Connective Tissue Growth Factor and Activation of Wnt Signaling in Rat Müller Cells

**FIGURE 7:**
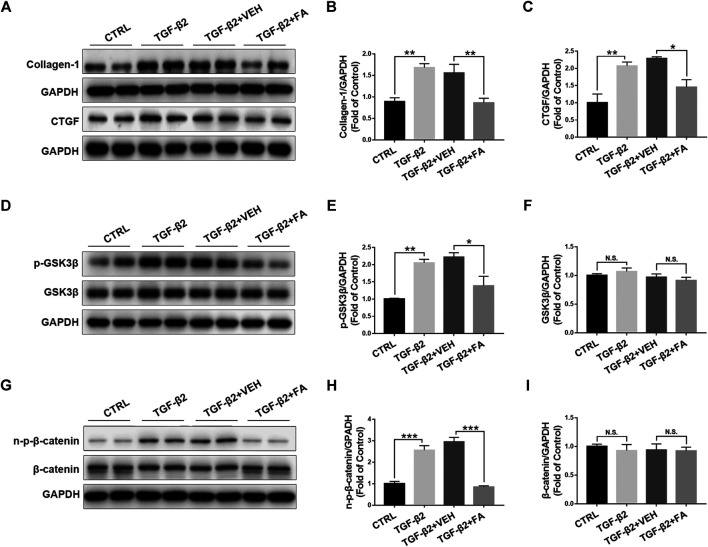
Fenofibrate inhibits TGF-β2–induced up-regulation of connective tissue growth factor (CTGF) and activation of Wnt signaling in rat Müller (rMC-1) cells. rMC-1 cells were treated with 5 ng/ml TGF-β2 for 2 h and then incubated with vehicle (VEH) and 200 μM fenofibric acid (FA) for additional 24 h. Cells were divided into 4 different groups: control cells (untreated), cells treated with TGF-β2 only, cells treated with TGF-β2 and vehicle, and cells treated with TGF-β2 and FA. The protein expressions of **(A,B)** collagen-1 and **(A,C)** CTGF in the four groups were determined by Western blot analysis and quantified by densitometry. Similarly, the protein expression of **(D,E)** p-glycogen synthase kinase 3 beta (GSK3β), **(D,F)** GSK3β, **(G,H)** non–p-β-catenin, and **(G,I)** β-catenin in the four groups were determined by Western blot analysis and quantified by densitometry (mean ± SEM, n = 6. N.S., non-significant; *p < 0.05, **p < 0.01 and ***p < 0.001).

To further test the effects of fenofibrate on inhibiting Müller cell activation and reducing CTGF expression, we incubated rMC-1 cells with TGF-β2 to induce Müller cell activation and fibrosis, and then treated the incubated rMC-1 cells with fenofibric acid (FA). FA is the active metabolite of fenofibrate administrated orally and used as a substitute for fenofibrate in cell experiments (Moran et al., 2014). The cell proliferation assay was performed in rMC-1 cells treated with different concentrations of FA. No toxic effect of FA was observed at the concentrations of 10, 50, 100, and 200 μM (Supplementary Figure S3). The incubation of TGF-β2 increased the protein expressions of collagen‐1 and CTGF in rMC-1 cells, while FA reversed the up-regulated collagen‐1 and CTGF (Figures 7A–C). Meanwhile, TGF-β2 up-regulated the protein levels of Wnt signaling components, p-GSK3β (Figures 7D,E), GSK3β (Figures 7D,F), non–p-β-catenin (Figures 7G,H), and β-catenin (Figures 7G,I), whereas FA decreased the protein levels of these Wnt signaling components (Figures 7D–I). Taken together, these results demonstrate that FA suppresses the expression of CTGF and Wnt signaling activation in cultured Müller cells.

### Fenofibrate Suppresses Subretinal Fibrosis in the Retina of Aged Very Low–Density Lipoprotein Receptor^*−/−*^ Mice

**FIGURE 8:**
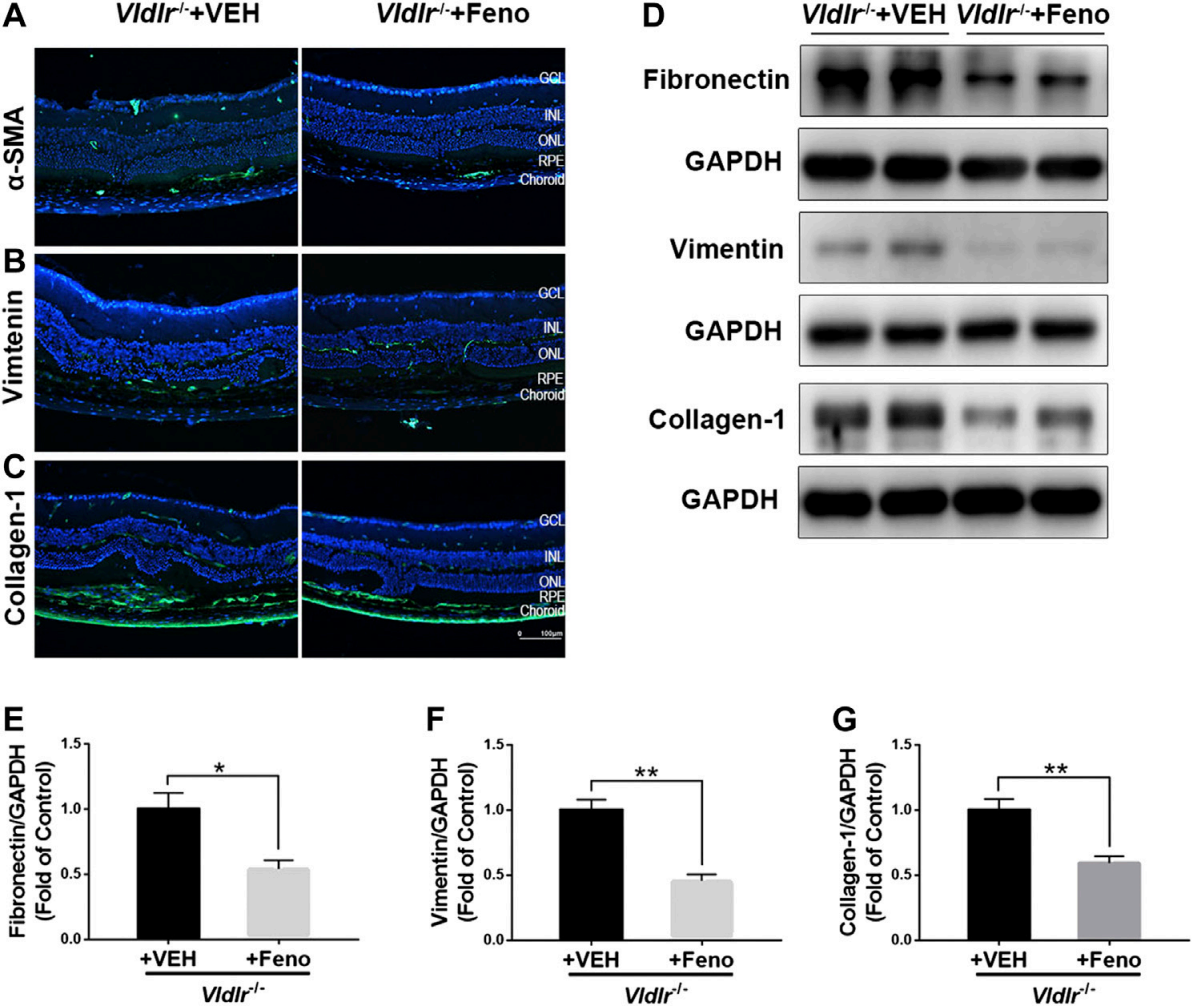
Fenofibrate suppresses subretinal fibrosis in aged very low–density lipoprotein receptor (*Vldlr*)^*−/−*^ mice. Representative images of immunofluorescence of **(A)** alpha-smooth muscle actin (α-SMA), **(B)** vimentin, and **(C)** collagen-1 in the eye of *Vldlr* ^*−/−*^ mice fed with normal chow (VEH) or fenofibrate chow (Feno). Protein levels of **(D,E)** fibronectin, **(D,F)** vimentin, and **(D,G)** collagen-1 were determined by Western blot analysis and quantified by densitometry (mean ± SEM; n = 4. *p < 0.05 and **p < 0.01).

The fibrotic changes in 3-month-old *Vldlr* ^*−/−*^ mice are accompanied with angiogenesis, which may represent the pathological changes of the early stage of nAMD. *Vldlr* ^*−/−*^ mice at the age of 6 months have vessel regression and subretinal fibrosis (Hu et al., 2008), which may recapitulate some features of the late stage of subretinal fibrosis. Thus, we used 7-month-old *Vldlr* ^*−/−*^ mice to further test the effects of fenofibrate on subretinal fibrosis. The results showed that fenofibrate reduced the immunofluorescence of the fibrotic markers α-SMA (Figure 8A), vimentin (Figure 8B), and collagen-1 (Figure 8C) in *Vldlr* ^*−/−*^ retinas. Protein levels of fibronectin (Figures 8D,E), vimentin (Figures 8D,F), and collagen-1 (Figures 8D,G) were significantly down-regulated in the retina of *Vldlr* ^*−/−*^ mice treated with fenofibrate compared with those treated with VEH. Taken together, these results indicated that fenofibrate has anti-fibrotic effects even in the late stage of nAMD with subretinal fibrosis in the context of *Vldlr* ^*−/−*^ mice.

## Discussion

Subretinal fibrosis is a major cause of vision loss in patients with nAMD (Mitchell et al., 2018). In the present study, we have identified the presence of subretinal fibrosis in the *Vldlr* ^*−/−*^ mouse, indicating *Vldlr* ^*−/−*^ mouse is a useful tool for studying retinal fibrosis. We have also found that fenofibrate suppresses subretinal fibrosis in *Vldlr* ^*−/−*^ mouse as well as Müller gliosis, suggesting the possible application of fenofibrate on subretinal fibrosis. Moreover, fibrotic TGF-β—Smad2/3 signaling and Wnt signaling were dramatically up-regulated in *Vldlr* ^*−/−*^ retinas, indicating the possible roles of these two pathways in subretinal fibrosis. Fenofibrate is capable of suppressing these two fibrotic pathways and their downstream CTGF, suggesting the mechanism by which fenofibrate suppresses subretinal fibrosis. Müller cells are identified to be a main source of CTGF, and fenofibrate inhibits Müller cell activation and reduces the expression of CTGF in *Vldlr* ^*−/−*^ retinas. Our study identifies the inhibitory effects of fenofibrate on subretinal fibrosis and demonstrates its molecular mechanism of action by suppressing fibrotic TGF-β—Smad2/3 signaling and Wnt signaling as well as their downstream CTGF expression, suggesting that fenofibrate could be a potential treatment for nAMD with subretinal fibrosis.

VLDLR is a lipid protein receptor that mediates the lipid metabolism and has other functions beyond the lipid metabolism (Hussain et al., 1999). *Vldlr* ^*−/−*^ mice display a unique angiogenesis pattern, in which new vessels originated from the inner layer of retinal vasculature grow into the avascular photoreceptor layer, and the pathological choroid vessels also grow into the avascular layer (Chen et al., 2007; Sun et al., 2017). The two vasculatures form retinal–choroidal anastomosis, which may recapitulate key features of a late stage of retinal angiomatous proliferation (RAP), a special type of nAMD (Chen et al., 2007; Hu et al., 2008). Thus, *Vldlr* ^*−/−*^ mouse was considered to be an animal model of nAMD. Hu et al. reported *Vldlr* ^*−/−*^ mouse is an animal model of RAP and found that staining of vimentin was localized at the retinal lesion sites of the *Vldlr* ^*−/−*^ mice at the age of 12 months (Hu et al., 2008). In this study, we specifically focused on the fibrotic changes in the retinal of *Vldlr* ^*−/−*^ mice and have evaluated several fibrotic makers, including collagen deposition, vimentin, fibronectin, α-SMA, and the fibrotic signaling pathways, TGF-β signaling and Wnt signaling. Meanwhile, we put forward the idea that *Vldlr* ^*−/−*^ mouse could be an animal model of subretinal fibrosis.

The laser-induced CNV model is a widely accepted and used animal model for nAMD. The procedure of laser surgery induces breakage of RPE and neovascularization and mimics the hallmark pathology in nAMD (Tobe et al., 1998). Laser-induced CNV usually causes tissue injury and injury-related angiogenesis, which may not reflect the real pathways as angiogenesis occurring in AMD with the age-related and chronic process. *Vldlr* ^*−/−*^ mouse is a model of RAP and considered as a naturally occurred nAMD model (Hu et al., 2008; Joyal et al., 2016), which presents the key feature of nAMD, such as angiogenesis, ruptured RPE, and fibrosis. Since *Vldlr* ^*−/−*^ mouse is a genetic animal model, which may be more related to the chronic pathologic changes of human AMD.

The TGF-β signaling pathway has been widely accepted as a major pathway that contributes to various tissue fibrosis such as renal fibrosis, pulmonary fibrosis, and hepatic fibrosis (Meng et al., 2016; Hu et al., 2018). TGF-β ligands bind to the receptor TGF-βR2, which phosphorylates TGF-βR1 and subsequently activates the downstream regulators, Smad2 or Smad3, which ultimately increases gene expressions and promotes ECM formation (Nakao et al., 1997). A recent study has showed that TGF-β—Smad2/3 signaling played crucial roles in fibrotic diseases of the eye (Saika et al., 2008). In this study, we found activation of TGF-β—Smad2/3 signaling in the retina of *Vldlr* ^*−/−*^ mice, indicating its roles on subretinal fibrosis.

Wnt signaling is an evolutionarily conserved signaling pathway that plays essential roles in organ and tissue development and adult tissue hemostasis (Steinhart and Angers, 2018). Wnt signaling plays pathological roles in multiple fibrotic disorders, such as renal fibrosis and pulmonary fibrosis (Burgy and Konigshoff, 2018). For example, previous studies have reported that nuclear β-catenin was accumulated in activated fibroblast in the idiopathic pulmonary fibrosis (IPF) (Konigshoff et al., 2008). It is also reported that Wnt signaling was activated in tubular epithelial cells, which contributed to renal interstitial fibrosis (He et al., 2009). In the present study, we have identified that Wnt signaling was up-regulated in *Vldlr* ^*−/−*^ retinas, which was consistent with previous studies (Chen et al., 2007; Chen et al., 2016). Activation of Wnt signaling in *Vldlr* ^*−/−*^ retinas indicated its possible involvement in subretinal fibrosis. In this study, fenofibrate suppressed the collagen deposition and the expressions of fibrotic markers vimentin, fibronectin, collagen-1, and α-SMA, indicating its therapeutic effects on subretinal fibrosis. Moreover, fenofibrate inhibited both Wnt signaling and TGF-β signaling, indicating the action of its mechanism by suppressing these two pathways.

The crosstalk between Wnt signaling and TGF-β—Smad2/3 signaling has been reported. For example, Liang Xu et al. reported that Wnt/β-catenin signaling was crucial for the transitions of fibroblasts to myofibroblasts and required for TGF-β signaling–induced proliferation of lung fibroblasts in pulmonary fibrosis (Xu et al., 2017). Another study showed that WNT-5A, a ligand for Wnt signaling, regulated TGF-β levels and controlled its profibrotic activities in liver fibrosis (Beljaars et al., 2017). A recent study also showed that Wnt ligand (Wnt-1/Wnt-5a) secretion was a necessary downstream step for TGF-β–mediated myofibroblast differentiation and myocardial fibrosis in experimental autoimmune myocarditis (Blyszczuk et al., 2017). In the present study, TGF-β2 induced the up-regulation of GSK-3β and β-catenin levels in rMC-1 cells, suggesting the possible crosstalk of Wnt signaling and TGF-β in the retina. Further study is warranted to investigate the details of interactions between these two signaling pathways in the fibrosis of the eye.

CTGF is a secreted matricellular protein that modulates multiple signaling pathways that contribute to the development of fibrosis, such as pathways involving cell adhesion, migration, proliferation, collagen synthesis, and ECM deposition (Ihn, 2002). CTGF was highly expressed in tissues of many fibrotic diseases, such as pulmonary fibrosis (Lipson et al., 2012) and hepatic fibrosis (Dessein et al., 2009). Studies have shown that CTGF was up-regulated in the human diabetic retina and involved in diabetic-induced basal lamina thickening of retinal capillaries (Kuiper et al., 2008). The clinical trial of F3019, a human monoclonal anti-CTGF antibody, demonstrated that the blockage of CTGF was safe and well tolerated in IPF and was effective in some patients with IPF (Raghu et al., 2016). In this study, we found the CTGF levels were up-regulated in *Vldlr* ^*−/−*^ retinas, suggesting the important roles of CTGF in subretinal fibrosis.

Müller cells were fibroblast-like cells in the retina and may play essential roles in fibrotic changes of retina (Friedlander, 2007). Immunostaining of CTGF and GS showed colocalization of CTGF and GS, suggesting that Müller cells are the source of CTGF in the context of *Vldlr* ^*−/−*^ retina. Recently, a study has also shown that Müller cells secret CTGF that may interact or activate other cells in the retina, and thus affecting the signaling transduction and cellular responses (Liu et al., 2020). For instance, CTGF actives fibroblasts, induces the expression of TGF-β, and increases the deposition of ECM proteins, which leads to tissue remodeling and fibrosis. Fenofibrate was capable of suppressing the secretion of CTGF by Müller cells, thus blocking CTGF-mediated communications between Müller cells and other cell types in the retina during the fibrosis process, further supporting that inhibition of Müller-secreted CTGF is the main mechanism by which fenofibrate inhibits subretinal fibrosis. Interestingly, studies have shown that CTGF acts as a downstream regulatory factor of the TGF-β—Smad2/3 signaling pathway and Wnt signaling. Therefore, the inhibition of these two pathways by fenofibrate may also down-regulate the expression of CTGF.

To further investigate the roles of fenofibrate in suppressing retinal fibrosis and Müller-secreted CTGF, cultured rat Müller cells were used as an *in vitro* model. Studies have showed that the vitreal concentration of TGF-β2 was correlated to the contractile effects of collagen gels in patients with proliferative DR and PVR (Kita et al., 2008). TGF-β2 promoted the epithelial-mesenchymal transition and ECM deposition in the retina (Cao et al., 2019). In this study, TGF-β2 induced collagen-1 and CTGF expression and activated Wnt signaling in cultured Müller cells. Fenofibrate suppressed fibrosis of Müller cells and inhibited the activation of Wnt signaling. In addition, the effects of fenofibrate on aged *Vldlr* ^*−/−*^ mice were evaluated; fenofibrate was capable of inhibiting subretinal fibrosis in aged *Vldlr* ^*−/−*^ mice, further supporting fenofibrate’s therapeutic effects on subretinal fibrosis.

In all, fenofibrate inhibited subretinal fibrosis by suppressing TGF-β—Smad2/3 signaling and Wnt signaling, inhibiting Müller cell activation and reducing the expression of CTGF in an animal model of nAMD, suggesting fenofibrate could be a potential treatment for nAMD with subretinal fibrosis.

## Data Availability Statement

The raw data supporting the conclusions of this article will be made available by authors under request.

## Ethics Statement

The animal study was reviewed and approved by the Experimental Animal Ethics Committee of Xiamen University.

## Author Contributions

QC conceived and designed the experiments, performed the experiments, analyzed the data and wrote the manuscript. NJ designed the experiments, performed the experiments, analyzed the data and wrote the manuscript. YZ designed the experiments, performed the experiments, analyzed the data. SY, XuL, XW and XiL performed the experiments. RZ and HC contributed reagents/materials. ZL conceived the experiments, analyzed the data and wrote the manuscript.

## Funding

This study was supported by grants from the National Science Foundation for Young Scientists of China (Grant NO. 31807795), National Key R&D program of China (Grant NO. 2018YFA0107302) and Natural Science Foundation of Fujian Province (Grant NO. 2019J01017).

## Conflict of Interest

The authors declare that the research was conducted in the absence of any commercial or financial relationships that could be construed as a potential conflict of interest.
