# Supplementary material for: Fenofibrate Inhibits Subretinal Fibrosis Through Suppressing TGF‐β—Smad2/3 signaling and Wnt signaling in Neovascular Age‐Related Macular Degeneration
Source: Front Pharmacol. 2020 Nov 17;11:580884. doi: 10.3389/fphar.2020.580884 (PMC7797782; doi:10.3389/fphar.2020.580884)
Supplement: Supplementary file 1 [file datasheet1.pdf]

Supplemental Figures and Figure Legends

Supplemental Figure 1. Collagen deposition at different ages of *Vldlr*$^{-/-}$ mice. Representative images of Masson staining of Collagen deposition in the lesion sites of *Vldlr*$^{-/-}$ mice at ages of 2 months, 3 months, 5 months and 8 months.

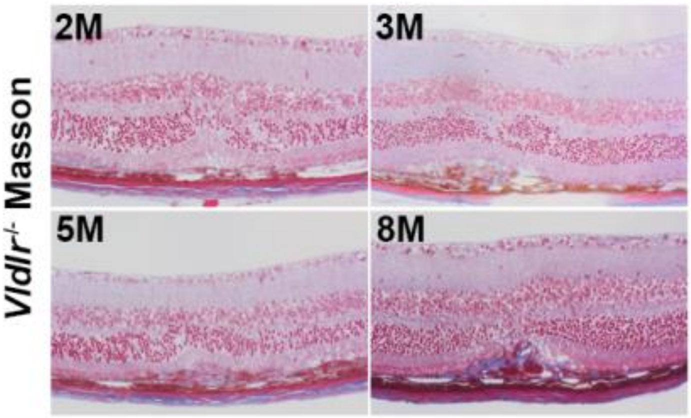

Supplemental figure 1

Supplemental Figure 2. Fenofibrate upregulates ACOX1 expression in the retina. (A, B) The protein expressions of ACOX-1 were measured in the retinas of WT mice and *Vldlr*$^{-/-}$ mice fed with control chow (*Vldlr*$^{-/-}$+VEH) or fenofibrate chow (*Vldlr*$^{-/-}$+Feno) by Western blot analysis and quantified by densitometry. (mean±SEM; n=8. N.S.= non-significant, ***p<0.001)

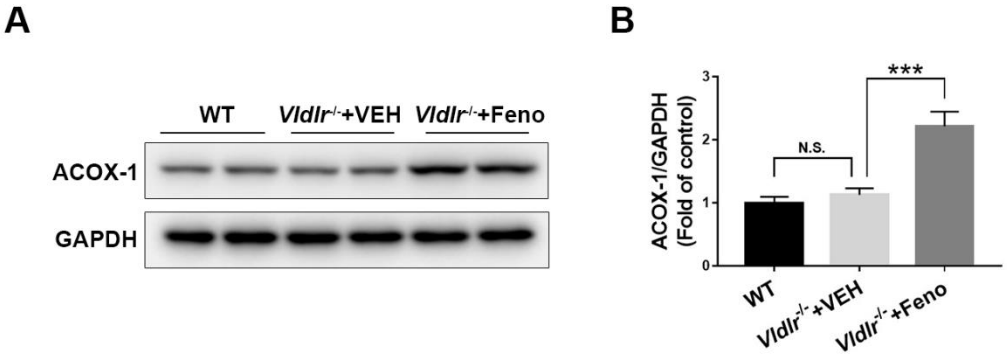

Supplemental figure 2

Supplemental Figure 3. Different concentrations of fenofibric acid (FA) on viabilities of rMC-1 cells. The cell proliferation assay was performed in rMC-1 cells treated with different concentrations of FA as shown in the figure. No toxic effect of FA was observed at the concentrations of 10, 50, 100, 200μM. (mean±SEM; n=3. **P<0.01, ***p<0.001)

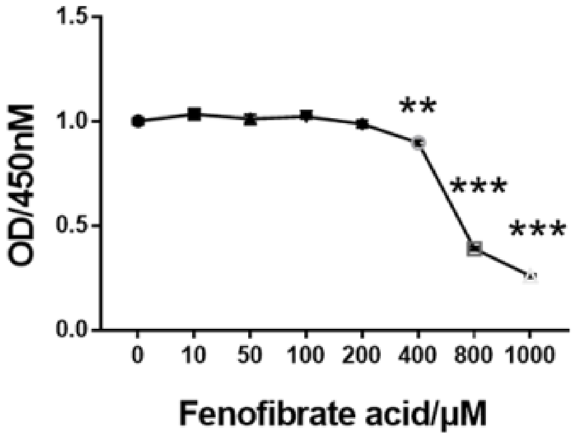

Supplemental figure 3
